# Cytotoxic T cells expressing the co-stimulatory receptor NKG2 D are increased in cigarette smoking and COPD

**DOI:** 10.1186/1465-9921-11-128

**Published:** 2010-09-24

**Authors:** Ester Roos-Engstrand, Jamshid Pourazar, Annelie F Behndig, Anders Blomberg, Anders Bucht

**Affiliations:** 1 Department of Public Health and Clinical Medicine, Division of Medicine, Umeå University, Sweden; 2 Department of Medicine, Division of Respiratory Medicine & Allergy, Umeå University, Sweden; 3 Swedish Defence Research Agency, Division of CBRN Defence and Security, Umeå, Sweden

## Abstract

**Background:**

A suggested role for T cells in COPD pathogenesis is based on associations between increased lung cytotoxic T lymphocyte (CD8^+^) numbers and airflow limitation. CD69 is an early T cell activation marker. Natural Killer cell group 2 D (NKG2D) receptors are co-stimulatory molecules induced on CD8^+ ^T cells upon activation. The activating function of NKG2 D is triggered by binding to MHC class 1 chain-related (MIC) molecules A and B, expressed on surface of stressed epithelial cells. The aim of this study was to evaluate the expression of MIC A and B in the bronchial epithelium and NKG2 D and CD69 on BAL lymphocytes in subjects with COPD, compared to smokers with normal lung function and healthy never-smokers.

**Methods:**

Bronchoscopy with airway lavages and endobronchial mucosal biopsy sampling was performed in 35 patients with COPD, 21 healthy never-smokers and 16 smokers with normal lung function. Biopsies were immunohistochemically stained and BAL lymphocyte subsets were determined using flow cytometry.

**Results:**

Epithelial CD3^+ ^lymphocytes in bronchial biopsies were increased in both smokers with normal lung function and in COPD patients, compared to never-smokers. Epithelial CD8^+ ^lymphocyte numbers were higher in the COPD group compared to never-smoking controls. Among gated CD3^+^cells in BAL, the percentage of CD8^+ ^NKG2D^+ ^cells was enhanced in patients with COPD and smokers with normal lung function, compared to never-smokers. The percentage of CD8^+ ^CD69^+ ^cells and cell surface expression of CD69 were enhanced in patients with COPD and smokers with normal lung function, compared to never-smokers. No changes in the expression of MIC A or MIC B in the airway epithelium could be detected between the groups, whereas significantly decreased soluble MICB was detected in bronchial wash from smokers with normal lung function, compared to never-smokers.

**Conclusions:**

In COPD, we found increased numbers of cytotoxic T cells in both bronchial epithelium and airway lumen. Further, the proportions of CD69- and NKG2D-expressing cytotoxic T cells in BAL fluid were enhanced in both subjects with COPD and smokers with normal lung function and increased expression of CD69 was found on CD8^+ ^cells, indicating the cigarette smoke exposure-induced expansion of activated cytotoxic T cells, which potentially can respond to stressed epithelial cells.

## Background

Chronic obstructive pulmonary disease, COPD, is characterized by a progressive airway obstruction and pulmonary inflammation. Studies have shown that inflammation in COPD occurs in central and peripheral airways as well as in the lung parenchyma [1,2]. Cigarette smoking is the major risk factor for the development of COPD and cigarette smokers constitute over 90% of COPD patients in developed countries [3]. In 2030, the WHO has predicted COPD to be the third leading cause of death worldwide. http://www.who.int. However, the mechanisms by which cigarette smoke induces COPD are still elusive.

The suggested role for T cells in the pathogenesis of COPD is based on the associations between airflow limitation and increased cytotoxic T lymphocytes (CD8^+^) in the airways and lung tissue [4-6]. Specifically, increased CD8^+ ^T cell numbers have been found in the airways of smokers in the early phase of COPD [5], as well as in sputum, lung tissue and BAL fluid from patients with established COPD [1,7,8]. The reason for this increase is still not clear but viral infections [9] and bacterial colonisation [10] have been suggested to provoke the cytotoxic T cell responses.

We have previously reported increased numbers of CD8^+ ^lymphocytes in the airway epithelium of subjects with COPD compared to smokers with normal lung function [11,12]. Moreover, compared to never-smokers, the expansion and activation of airway CD8^+ ^lymphocytes, in terms of increased expression of CD69, HLA-DR and CD25, have been demonstrated in smokers without a clinical diagnosis of COPD (12). CD69 is an early activation marker expressed on T, B and Natural Killer (NK)-cells. CD69 is an inducible cell surface glycoprotein involved in lymphocyte proliferation and signal transduction [13]. On the other hand, HLA-DR is a late activation marker, upregulated 48-60 hours after T cells receptor (TCR) stimulation, and is regarded as a more general marker of activated T-cells. High expression of CD25 is associated with the presence of regulatory T cells with down modulating action on inflammatory reactions. Our previous data indicate that cigarette smoke exposure *per se *would trigger the activation of CD8^+ ^T cells. A possible mechanism could be induction of oxidative stress by components in the tobacco smoke [12]. However, the expansion of activated cytotoxic T cells persisted in the airways of COPD patients more than five years after smoking cessation [12], suggesting a role for cytotoxic T cells also in established COPD.

Epithelial cells undergoing stress are compromised in function and are normally removed in order to control inflammation and promote cellular repair. Multiple mechanisms for the detection and elimination of stressed cells have been described, including immune cell activation[14]. As a consequence of tobacco smoke exposure, injured or dead epithelial cells may be increased due to epithelial injury combined with impaired phagocytotic function [15]. A mechanism that may provide a link between epithelial cell stress and immune cell activation in the lungs involves the recognition of stressed epithelial cells by the NK cell group 2 D (NKG2D) receptor. The NKG2 D receptors are constitutively expressed almost exclusively on some cytotoxic T lymphocytes (NK cells, NK T cells and γδ^+ ^T cells), whereas the NKG2 D receptor expression on CD8^+ ^T cells is induced upon activation [16]. The mechanism by which the immune system recognizes injured or stressed epithelial cells may represent a critical pathway involved in tissue repair and remodeling processes required for the preservation of pulmonary tissue and the gas exchange function of the lung [17]. Recognition of NKG2 D ligands by NKG2 D also appears to play a role in the pathogenesis of diseases that are associated with autoimmune conditions [16]. Lee *et al *have suggested that emphysema is an autoimmune disease characterized by correlation with anti-elastin antibody and T-helper type 1 response, providing a putative link of emphysema severity to adaptive immunity against specific lung antigens [18].

Cytotoxic T cells can recognise stressed or infected cells through the binding of NKG2 D to the MHC class 1 chain-related (MIC) molecules A and B, expressed on the surface of the target cells [19]. Normally, MIC proteins are constitutively expressed at a low degree on lung epithelial cells and are upregulated upon cell activation or stress in smokers, possibly as a consequence of oxidative stress [19]. Cell activation mediated by NKG2 D binding to MIC induces proliferation, survival, activation marker expression, cytokine production and cytotoxicity in CD8^+ ^T cells, NK-cells and γδ^+ ^T cells [20]. In CD8^+ ^T cells, NKG2 D is a potent co-stimulatory receptor to TCR-CD3 complex-dependent T cell activation [21].

The importance of interactions between NKG2 D and MIC molecules in COPD is not well understood. However, Borchers *et al *have recently reported NKG2D-mediated cytotoxic T cell activation in mice after exposure to cigarette smoke [18]. NKG2 D ligand over-expression induced emphysema in transgenic mice and NKG2 D ligands were abnormally expressed in the pulmonary epithelium of both animals exposed to cigarette smoke and patients with COPD, implying a role in the pathogenesis of COPD [19]. These authors also postulated that sustained expression of NKG2 D ligands would lead to disruption of the alveolar architecture by cytotoxic T lymphocyte-mediated apoptosis of pulmonary epithelial cells. The aim of the present study was therefore to further investigate the activation of cytotoxic T cells in stable COPD, as well as the importance of the NKG2 D receptor and MIC ligands in smokers and COPD patients.

## Methods

### Subjects

Seventy-two subjects participated in this study, 35 patients with moderate to severe COPD according to GOLD-criteria (FEV_1 _35-80% of predicted), 21 healthy volunteers with no smoking history (NS) and 16 smokers with normal lung function (S). Subject demographics are given in table 1. Of the COPD patients, 16 were current smokers and 19 were ex-smokers, with smoking cessation more than five years prior to inclusion. COPD subjects and smokers with normal lung function had a smoking history of at least ten pack-years (one pack year equals to 20 cigarettes/day). Current smokers were not allowed to smoke for at least 12 hours prior to bronchoscopy. Both COPD-groups had significantly reduced lung function described as FEV_1_/FVC% ratio and FEV_1_% of predicted post-bronchodilatation compared to both smokers with normal lung function (p < 0.001 and p < 0.001) and never-smokers (p < 0.001 and p < 0.001).

**Table 1:** Demographics and spirometry values

	Never-smokers n = 21	Smokers n = 16	COPD Ex-smokers n = 19	COPD Smokers n = 16
Male:Female	11:10	7:9	13:6	4:12
Age	66 ± 5.2	61 ± 6.8	68 ± 5.8	64 ± 5.5
Smoking(pack years)	0 (0-0)	31 (21-42)	35 (18-42)	29 (24-48)
COPD stage^#^	NA	NA	2 and 3	2 and 3
FEV_1_/FVC %	78 (76-82)	79 (77-83)	55 (49-64)*	60 (57-63)*
FEV_1 _% of predicted	101 (90-116)	110 (99-119)	46 (36-53)*	54 (39-68)*

Data are shown as mean±standard deviation for age, median and inter quartile range for all others. FEV_1_: Forced expiratory volume in one second, measured post bronchodilatation; FVC: Forced vital capacity. # according to GOLD standard [3]. NA: not available. *indicate significant difference compared to smokers with normal lung function and never-smokers (p < 0.001).

In a sub study, flow cytometry analyses were performed in 9 patients with moderate to severe COPD, 9 healthy volunteers with no smoking history (NS) and 14 smokers with normal lung function (S). Of the COPD patients, 5 were current smokers and 4 were ex-smokers.

The COPD patients had received no treatment with inhaled corticosteroids for at least four weeks prior to the start of the study. Neither long-acting β_2_-agonists nor long-acting anti-cholinergic drugs were allowed within two weeks prior to bronchoscopy. Short-acting β_2_-agonists and/or anti-cholinergic drugs were used on demand. All the subjects were non-atopic and free from symptomatic respiratory infection within a six week-period prior to and during the study. They had no history of chronic bronchitis or frequent exacerbations. No anti-inflammatory drugs, such as non-steroidal anti-inflammatory drugs or oral steroids or any additional intake of vitamin C or E were allowed. Informed consent was obtained from all volunteers after verbal and written information and the study was approved by the local Ethics Committee at Umeå University, Sweden, and performed according to the declaration of Helsinki.

### Methods

#### Spirometry

Dynamic spirometry variables (FVC and FEV_1_) were determined post bronchodilatation with 1 mg of terbutaline, using a Vitalograph spirometer (Vitalograph Ltd. Buckingham, UK). At least three satisfactorily performed and well-co-operated measurements were carried out, according to the recommendations of the American Thoracic Society [22].

#### Bronchoscopy

Atropine was given subcutaneously before bronchoscopy and topical anaesthesia of the airways was obtained using lidocaine. The subjects were examined in the supine position using an Olympus BF IT160 video bronchoscope (Olympus, Tokyo, Japan). Bronchial wash (BW) was performed by infusing two aliquots of 20 ml of sterile sodium chloride (NaCl), pH 7.3 at 37°C that were gently sucked back after each infusion and pooled into a tube placed in iced water. Bronchoalveolar lavage (BAL) was performed by infusing three aliquots of 60 ml of sterile sodium chloride (NaCl) in either the middle or lingula lobe. Four to six endobronchial mucosal biopsies were collected, from the proximal airway carinae of the contra-lateral lung. Endobronchial biopsies and recovered fluids were immediately transported to the laboratory for analysis.

#### Processing and staining of bronchial biopsies

The endobronchial mucosal biopsies were immediately fixed in ice cold acetone with the inclusion of the protease inhibitors phenylmethylsulphonyl fluoride (2 mM) and iodoacetamine (20 mM). The biopsies were subsequently processed into glycol methacrylate (GMA) resin according to a previously described protocol [23]. The GMA blocks were stored in air tight containers at -20°C until the staining procedure was initiated. In order to detect mucus secreting cells (goblet cells) in the epithelium, two sections from each subject were stained with Alcian blue. Immunohistochemical staining with monoclonal antibodies (MoAbs) against CD3, CD4, CD8 (DAKO, Glostrup, Denmark), NKG2 D, MICB (R & D, Abingdon, UK), and a polyclonal antibody against MICA (R & D, Abingdon, UK) was performed as described in detail elsewhere [24]. Briefly, 2-μm sections were treated to inhibit endogenous peroxidases by applying a solution of 0.3% hydrogen peroxide in 0.1% sodium azide. Additionally, non-specific antibody binding was blocked by the use of Dulbecco's minimal essential medium containing 10% foetal calf serum and 1% bovine serum albumin. Undiluted blocking medium was applied for 30 minutes and then poured off, whereupon mouse MoAbs and goat polyclonal antibodies were applied and incubated for 16-20 h at room temperature. Biotinylated rabbit anti-mouse immunoglobulin F(ab')_2 _(Dako, Glostrup, Denmark) or swine anti-goat immunoglobulin was added to each section and incubated for 2 h, and subsequently a complex of streptavidin-biotin-horseradish and peroxidase (Dako) was added for 2 h. After rinsing in TBS, aminoethyl carbazole (AEC) in distilled water and hydrogen peroxide were used to induce a red colour, in this manner marking positive immunoreactions. 3.3-diaminobenzidine (DAB) diluted in distilled water and hydrogen peroxide induced a brown colour for positive immunoreactions. All sections were counterstained with Mayer's haematoxylin. Primary antibody was omitted on sections serving as negative controls. Two stained sections from each participant were estimated with respect to epithelium quality, and the best of these was used for quantifying positively stained epithelial cells.

#### Quantification of staining in mucosal biopsy specimens

The epithelium was defined as the intact area above the basement membrane. The total length was calculated by use of computer assisted image analysis (Qwin, Leica Q500IW; Leica, Cambridge, UK). Quantification of positively stained cells was performed using a light microscope. The number of positively stained cells was expressed as cells per millimeter of epithelium as, previously described [24]. Quantification of MIC antigens in the bronchial epithelium was performed on sections developed with DAB as the substrate. The epithelium expression was quantified and expressed as the percentage of epithelial area with positive immunostaining as compared with the total epithelial area. Quantification of NKG2 D antigens was performed on sections developed with AEC as the substrate.

**Figure 1:**
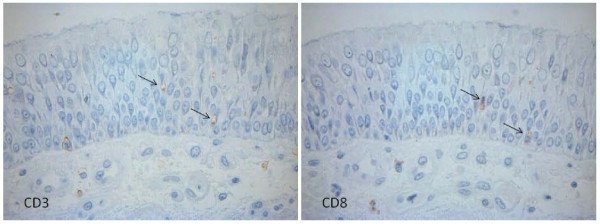
**Immunohistochemistry staining of serial sections for CD3 and CD8**. The arrows indicate positive cell staining.

Positive staining for CD3^+^, CD4^+ ^and CD8^+ ^lymphocytes revealed a ring staining pattern (figure 1).

#### Cell preparation and MIC analyses

The chilled bronchial wash (BW) and bronchoalveolar lavage (BAL) were processed by filtrated through a nylon filter (pore diameter 100 μm, Syntab Product AB, Malmö, Sweden) and centrifuged (400 g, 15 minutes, at 4°C). The cell pellet was resuspended in PBS. The total number of cells was counted and adjusted to a final concentration of 10^6 ^cells/ml.

Cytocentrifuged specimens with 50 μl suspended cells, from 10^6^/ml, were prepared using a Cytospin 2^® ^at 450 rpm for 5 minutes (Shandon Southern Instruments Inc., Sewikly, PA, USA). Slides were stained according to May-Grünwald Giemsa and 500 cells per slide were counted for standard cell differential counts.

The measurement of soluble MICA and MICB in BAL and BW supernatant was performed by commercially available Elisa kits, (R & D Systems, Abingdon United Kingdom.).

#### Flow cytometry analysis

Lymphocyte subsets in BAL were determined by flow cytometry. BAL cells were centrifuged and diluted to a final concentration of 10^6 ^cells/ml. For each test, 10 μl of antibody solution was added to 200 μl of cell suspension and allowed to bind for 30 minutes at 4°C in darkness. Red blood cells were lysed with 2 ml FACS™Lysing solution (Becton Dickinson Immunocytometry Systems, San Jose, CA, USA) for 10 minutes at room temperature and the remaining cells were washed by adding PBS to the tubes and centrifuged at 4°C for 10 minutes, 300 g. This washing procedure was performed twice. Cells were then fixed with 500 μl CellFIX™(Becton Dickinson Immunocytometry Systems, San Jose, CA, USA) before analysis using a FACScan™(Becton Dickinson) flow cytometer. Up to 10,000 total events were collected per sample. The lymphocyte population was gated based on their physical characteristics in a region, according to their characteristic forward scatter (FCS) and side scatter (SSC) profiles. To obtain CD3^+^, CD8^+ ^and NKG2D^+ ^cells, the cells were stained with peridinin chlorophyll protein complex (PerCP) conjugated anti-human CD3, fluorescein isothiocyanate (FITC) conjugated anti-human CD8 and phycoerytrin (PE) conjugated anti-human NKG2 D in the same test tube. The percentage of different cell types was counted out of gated CD3^+ ^lymphocytes. Source of antibodies for flow cytometry was Becton Dickinson Immunocytometry Systems, San Jose, CA, USA, except for NKG2 D which was provided by R & D, Abingdon, UK.

### Statistical analysis

Flow cytometry data were acquired and analysed using CellQuest Software (Becton Dickinson, San Jose, CA, USA). Statistical comparisons between the three groups were carried out using Kruskal-Wallis test and a p-value of less than 0.05 was considered significant. If the Kruskal-Wallis test indicated significance, the Mann-Whitney U-test was used for post-hoc analysis for comparison between two groups, with corrections of p-values according to Bonferroni (a p-value less than 0.017 was considered significant). In order to distinguish between COPD and smoking-related effects, a subgroup analysis was carried out within the COPD group. The ex-smoking COPD group was compared to both the smoking COPD group and the never smoking group, using Mann-Whitney U-test. Here, a p-value of less than 0.05 was considered significant.

## Results

### Immunohistochemistry analysis of endobronchial mucosal biopsies

**Figure 2:**
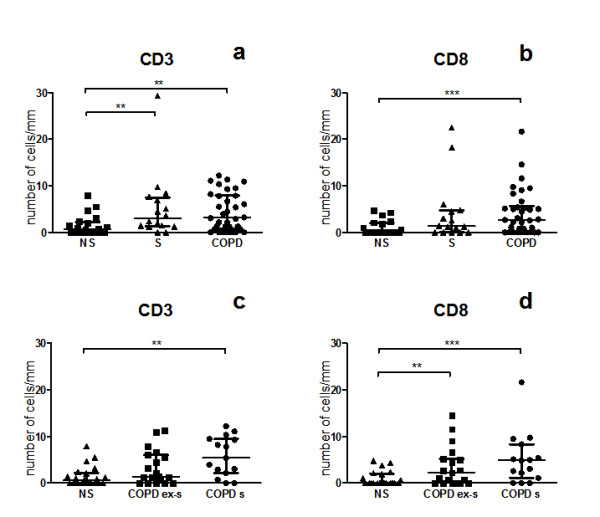
**Immunohistochemistry analysis of bronchial epithelium in never-smokers (NS), smokers with normal lung function (S), and COPD subjects (a and b)**. COPD subjects are further divided into ex-smokers (ex-s) and smokers (s) (c and d). CD3 and CD8 are given as number of cells per mm epithelium. Significance levels are noted as ** p < 0.01, *** p < 0.001. Data are given as median and IQR.

**Figure 3:**
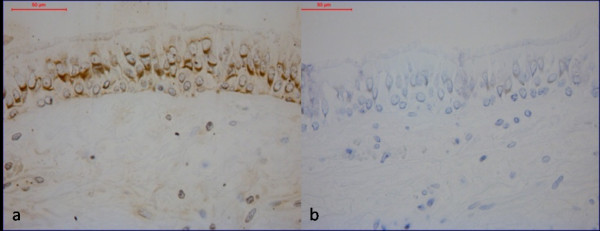
**Immunohistochemistry analysis of MIC A (a) and negative control (b)**. Positively stained cells are indicated by the brown colour (DAB).

**Figure 4:**
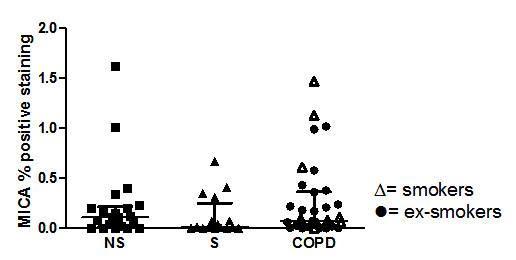
**The expression of MICA in epithelium of never-smokers (NS), smokers with normal lung function (S) and COPD**. Values are given in percent positive staining in bronchial epithelium. Among the COPD group (black circle) indicates ex-smokers, whilst (white triangle) indicates smokers. Data are given as median and IQR

The number of epithelial CD3^+ ^lymphocytes was increased in both smokers with normal lung function (3.00; 1.27-7.48 cells/mm^2^) (median; inter quartile range) and in the COPD group (3.14; 0.76-7.94), compared to never-smokers (0.65; 0.00-2.22) (p = 0.006 and p = 0.005 respectively), (figure 2). Epithelial CD3^+ ^lymphocyte numbers were similar in COPD subjects and in smokers with normal lung function. Epithelial CD8^+ ^lymphocyte numbers were higher in the COPD group (2.72; 0.00-5.69, p = 0.001) compared to the never-smoking controls (0; 0.99-2.01), but without a significant difference compared to smokers with normal lung function (1.34; 0.00-4.71). There was no difference in epithelial CD4^+ ^cells or MICA expression between the three groups, (figure 3 and 4). Neither MICB nor NKG2 D expression was detectable in bronchial tissue. Tonsil tissue acted as positive control for the MICB and NKG2 D stainings.

### Differential cell counts in BALF

Smokers with normal lung function had increased number of total leukocytes in BALF compared to never-smokers (p < 0.001), whereas a non significant increase was noted compared to subjects with COPD (p = 0.03). Among leukocytes, the number of macrophages was increased in smokers with normal lung function compared to never smokers (p < 0.001) with a trend towards a significant increase compared to COPD subjects (p = 0.02) (table 2). In smokers with normal lung function, the numbers of neutrophils were increased compared to never smokers (p = 0.007), whereas mast cell numbers were increased compared to COPD subjects (p = 0.003).

**Table 2:** Differential cell counts of white blood cells in BAL fluid, given in number cells/ml*10^4^.

	Never smokers (NS) n = 21	Smokers (S) n = 16	COPD n = 33	p	COPD ex-smokers n = 19	COPD smokers n = 14	p
**Total leukocytes**	19 (13-27)	40 (31-50)	25 (17-30)	**P < 0.001 **NS vs S **P = 0.03 **S vs COPD	18 (15-26)	29 (25-45)	**P = 0.02 **COPD ex-svs COPD s

**Macrophages**	16 (11-22)	37 (29-47)	22 (14-28)	**P < 0.001 **NS vs S**P = 0.02 **S vs COPD	15 (11-21)	27 (24-40)	**P < 0.001 **COPD ex-svs COPD s

**Neutrophils**	0.17 (0.08-0.29)	0.47 (0.24-0.98)	0.26 (0.06-0.5)	**P = 0.007 **NS vs S	0.28 (0.07-0.6)	0.19 (0.06-0.5)	NS

**Lymphocytes**	1.7 (0.9-2.7)	2.3 (1.4-3.6)	1.4 (0.90-3.0)	NS	2.0 (1.1-3.3)	1.3 (0.58-1.9)	NS

**Eosinophils**	0.06 (0-0.16)	0.05 (0-0.23)	0.12 (0.03-0.46)	NS	0.05 (0.01-0.35)	0.21 (0.05-0.62)	NS

**Mast cells**	0.01 (0.0-0.03)	0.03(0.005-0.04)	0.0 (0.0-0.01)	**P = 0.003 **S vs COPD	0.005 (0.00-0.01)	0.0 (0.0-0.008)	NS

To examine whether the difference in airway inflammation between COPD patients and smokers with normal lung function was due to smoking habits, the COPD patients were divided into current smokers and ex-smokers. The results of this subgroup analysis showed that smoking COPD patients had increased numbers of BAL macrophages (p < 0.001) compared to ex-smoking COPD patients (table 2).

### Analysis of T cells subpopulations in BALF

**Figure 5:**
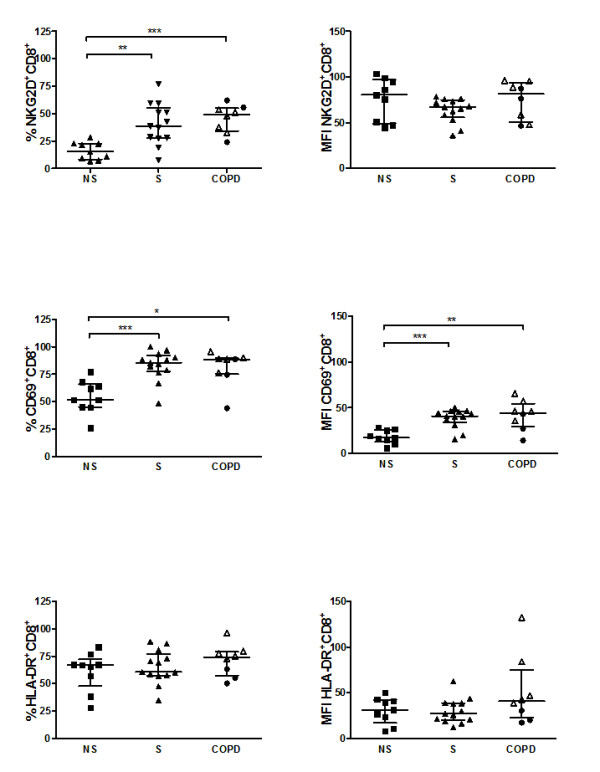
**Flow cytometry analyses of BAL T cells in never-smokers (NS), smokers with normal lung function (S) and COPD**. Data are given as percent and median fluorescent intensity (MFI) of gated CD3^+ ^cells. Within the COPD group, ex-smokers (black circle) and current smokers (white triangle) are indicated. A p-value below 0.017 is considered significant. Significance levels are noted as * p < 0.017, ** p < 0.01, *** p < 0.001. Data are given as median and IQR.

The percentage of CD8^+ ^NKG2D^+ ^cells among gated CD3^+^cells was enhanced in patients with COPD and smokers with normal lung function, compared to never-smokers (p = 0.001 and p = 0.002 respectively). The percentage of CD8^+^CD69^+ ^cells among gated CD3^+^cells was enhanced in patients with COPD and smokers with normal lung function compared to never-smokers (p = 0.012 and p = 0.001 respectively). Also, CD69 median fluorescence intensity was increased on CD8^+ ^T cells in COPD subjects and smokers with normal lung function compared to never-smokers (p = 0.007 and p = 0.001 respectively) (figure 5). There were no differences in median fluorescence intensity in NKG2 D or HLA-DR on CD8^+ ^T cells.

### Analysis of soluble MIC

**Figure 6:**
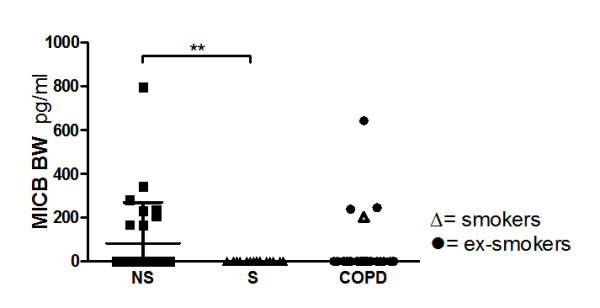
**Analysis of sMICB in bronchial wash (BW) of never-smokers (NS), smokers with normal lung function (S) and COPD by ELISA**. Values are given as pg/ml. Within the COPD group, ex-smokers (black circle) and current smokers (white triangle) are indicated. A p-value below 0.017 is considered significant. Significance levels are noted as ** p < 0.01. Data are given as median and IQR

Soluble MICB (sMICB) was undetectable in BW from smokers with normal lung function, whereas in never-smokers sMICB was detectable (83; 0.0-269 pg/ml, p = 0.002) (figure 6).

MICA was not detectable in either BW or BAL.

## Discussion

We have demonstrated increased numbers of CD8^+ ^T cells in the bronchial epithelium of subjects with COPD. These data are in concordance with previous studies of T cells in the bronchial epithelium and BALF from COPD patients performed by us and others [5,7,8,11,12]. In addition, we now report that the cytotoxic T cells in BALF of smokers with normal lung function and COPD patients express high levels of the co-stimulatory receptor NKG2 D and the early cell activation marker CD69. These data imply that cytotoxic T cells recruited to the airways of both COPD subjects and smokers without clinical symptoms of COPD are highly activated and can respond to stressed and injured epithelial cells.

The primary function of CD8^+ ^T cells is to recognize and kill cells expressing ligands stimulating cytotoxicity. They kill target cells by inducing apoptosis through secretion of perforin, granzyme, and by Fas/Fas ligand (FasL) interactions. An inverse relationship is reported between CD8^+ ^T lymphocytes and lung function in COPD [5] and that the extent of lung emphysema is associated with the number of T lymphocytes in lung parenchyma [25]. Further, several studies have shown that the numbers of CD8^+ ^T lymphocytes are increased in central and peripheral airways, also in emphysematous subjects [2,5,26,27]. Altogether, these findings support a role for cytotoxic T cells in the development of lung emphysema and COPD. Morissette *et al *did reported, however, no difference in expression of perforin, granzyme B and FasL of CD8^+ ^T lymphocytes in peripheral blood in emphysematous subjects [28], supporting the notion that CD8^+ ^T cells are not activated in the circulation of smokers and subjects with COPD. Despite data on increased T lymphocytes in emphysema and COPD, few studies have described their state of activation or function. It has been hypothesized that COPD have an autoimmune component [29] and Lee et al have reported that emphysema is characterized by an association of antielastin antibody and T helper 1 response [18]. In the present study we were not able to support a specific autoimmune component in COPD, since no differences were observed in the studied immune cell populations between COPD patients and smokers without clinical disease.

In this study, we detected the expansion of CD8^+^CD69^+ ^cells and the increased expression of the cell surface receptor CD69 on CD8^+ ^T cells in both COPD and smokers with normal lung function, compared to never-smokers. This finding is in line with our previously published data, supporting the hypothesis that CD8^+ ^T cells are highly activated in COPD but also in smokers without clinical symptoms of COPD [12]. CD69 is a constitutively expressed early activation marker and acts as a co-stimulatory molecule for T cell activation and proliferation, within one or two hours after TCR engagement [13]. It has also been shown that expression of CD69 is higher on sputum -lymphocytes than on peripheral blood cells [30]. The precise role for CD69 in immunity has not been elucidated owing to the absence of a known ligand and adequate *in vivo *models to study its physiological function [31]. In contrast to the data presented in this study, we previously reported that also HLA-DR is increased on CD8^+ ^cells in BALF [12]. The divergent results are due to differential gating procedures when performing analysis of flow cytometry data, i.e. in this study cytotoxic T cells were identified by using both anti-CD3 and anti-CD8 antibodies, while in the previous study only anti-CD8 antibodies were used to define the cytotoxic cells. Therefore, it is likely that the observed increase of HLA-DR in COPD and smokers with normal lung function was related to non-T cells with CD8 expression, such as small mast cells, macrophages or dendritic cells.

The NKG2 D ligands, MICA and B, are stress-inducible and primarily expressed on epithelial cells [32]. In spite of the large numbers of subjects included in the present study, we did not find any statistical difference when it comes to the NKG2 D ligand MICA in the bronchial epithelium. We hypothesize that the bronchial epithelium is not activated in terms of MICA or MICB, in our subjects with stable COPD. So far, only one published study has investigated the NKG2 D receptor and MIC ligands in COPD. In endobronchial biopsies and peripheral lung resection tissue from patients with lung cancer, Borchers *et al *found significantly increased expression of MICA in current smokers with COPD, compared to never smokers and former smokers with COPD. In that study, however, the COPD patients also suffered from lung cancer, which hypothetically may have influenced the MIC A expression, as NKG2D/NKG2 D ligand interactions also play a role in immune recognition of tumors [33]. The discordant result can also be due to differences in subjects medication, since inhaled steroid treatment was given to the patients in the study by Borchers. In our study, the subjects received no treatment with inhaled corticosteroids during at least four weeks prior to study start. Neither long-acting β_2_-agonists nor long-acting anti-cholinergic drugs were allowed within two weeks prior to bronchoscopy. All patients included in our study were in stable state of COPD, i.e. without any infectious exacerbations.

We did not detect an upregulation of MICA in bronchial biopsies from smokers or COPD subjects, and MICB was undetectable in all samples. Since the MIC proteins can be shedded from the epithelial cells into the alveolar space, we also analysed soluble MIC (sMIC) released to bronchial wash and BAL fluid. We observed that sMICB was decreased or undetectable in BW of smokers with normal lung function and nearly all subjects with COPD, when compared to never-smokers. This observation might be explained by adsorption of MICB by the large number of NKG2D-expressing cells in the airways of these individuals, or alternatively by the interference of cigarette smoking on the detection of sMICB in BW.

It should, however, be noted that there were no statistically significant increase in total lymphocyte numbers in our group of COPD patients, only the distribution of T cell subsets within the lymphocyte population was changed. It was also evident that subjects with stable COPD, did not display any increase in neutrophils or macrophages in BAL fluid. When sub-dividing the patients in current smoking and non-smoking, it was clearly demonstrated that expansion of macrophages in airways was associated with smoking rather than COPD, confirming our previously published data [12]. This is consistent with a primary role of macrophages in inflammatory responses to noxious particles and gases, e.g. components in tobacco smoke.

## Conclusions

In summary, we demonstrated an increased numbers of cytotoxic T cells in COPD, in both bronchial epithelium and airway lumen. CD69- and NKG2D-expressing T cells in BAL fluid were enhanced in both subjects with COPD and smokers with normal lung function, indicating that cigarette smoke exposure triggers the expansion of activated cytotoxic T cells, possibly by responding to injured epithelial cells. The cytotoxic T cells remained activated years after smoke cessation of COPD patients, implicating a role of these in the chronicity of COPD.

## Abbreviations

AEC: Aminoethyl carbazole; BAL: Bronchoalveolar lavage; BALF -Bronchoalveolar lavage fluid; BW -Bronchial wash; COPD: Chronic obstructive pulmonary disease; DAB: Diaminobenzidine; FEV_1: _Forced: expiratory volume in one second; FVC: Forced vital capacity; GMA: Glycol methacrylate; GOLD: Global Initiative for Chronic Obstructive Lung Disease; HLA-DR: Human Leukocyte Antigen DR; MICA: MHC class 1 chain-related (MIC) molecules A; MICB: MHC class 1 chain-related (MIC) molecules B; NKG2 D: Natural Killer cell group 2D; NS: Healthy volunteers with no smoking history; S: Smokers with normal lung function; WHO: World Health Organization

## Competing interests

The authors declare that they have no competing interests.

## Authors' contributions

ERE was responsible for flow cytometry and immunohistochemistry analyses, data and statistical analyses and manuscript preparation. JP was supervising flow cytometry and immunohistochemistry. AFB took part in patient recruitment, bronchoscopies and manuscript preparation. ABl was responsible for study design, patient recruitment, bronchoscopies and manuscript preparation. ABu was responsible for study design and manuscript preparation. All authors read and approved the final manuscript.
